# Factors influencing delayed clearance of high dose methotrexate (HDMTX) in pediatric, adolescent, and young adult oncology patients

**DOI:** 10.3389/fonc.2023.1280587

**Published:** 2023-10-26

**Authors:** Ema Mosleh, Stacy Snyder, Ningying Wu, Daniel N. Willis, Rema Malone, Robert J. Hayashi

**Affiliations:** ^1^ Department of Pediatrics, Washington University School of Medicine, St. Louis, MO, United States; ^2^ Biostatistics Shared Resource, Division of Public Health Sciences and Siteman Cancer Center, Department of Surgery, Washington University School of Medicine, St. Louis, MO, United States; ^3^ Division of Pediatric Hematology/Oncology, St. Louis Children’s Hospital, St. Louis, MO, United States

**Keywords:** methotrexate, HDMTX, supportive care, delayed clearance, nephrotoxicity, adverse effect, pediatric cancer, AYA

## Abstract

**Purpose:**

To identify modifiable risk factors associated with prolonged clearance of methotrexate in pediatric, adolescent, and young adult (AYA) oncology patients receiving high dose methotrexate (HDMTX).

**Design/Method:**

A single institution, retrospective chart review of patients receiving HDMTX between 2010-2017. Patients had a diagnosis of either leukemia or osteosarcoma. Data included demographics, concurrent intravenous (IV) medications, IV fluids (IVF) administered, urine output (UO), and rises in serum creatinine (RSC) reflective of renal toxicity (RT). Outcome measures included 1) delayed targeted MTX clearance (DC), 2) actual time to clearance (TTC) and 3) length of stay (LOS).

**Results:**

Data from 447 HDMTX administrations were analyzed. The sample consisted of 241 (54%) osteosarcoma encounters, and 206 (46%) leukemia encounters, with an average patient age of 12.7 years. Multivariate analysis showed that DC was associated with the diagnosis of leukemia (OR 7.64, p <.0001), and less UO on day 1 (OR 0.76, p=0.005). Increased TTC was associated with increasing age (RR 1.02, p<0.0001), higher 24-hour MTX levels (RR 1.001, p=0.012) and 48-hour MTX levels (RR 1.02, p<0.0001), RT (RR 1.004, p<0.0001), use of IV lorazepam (RR 1.08, p=0.001) and IV metoclopramide (RR 1.08, p<0.001) both on day 3. Like TTC, LOS was affected by MTX levels at 24 (RR 1.001, p=0.025) and 48 hours (RR 1.03, p<0.0001), RT (RR 1.006, p<0.0001), total IV medications on day 3 (RR 1.042, p<0.0001), and the use of leucovorin on day 2 (RR 0.93, p=0.002).

**Conclusion:**

Multiple modifiable risk factors were identified which can be leveraged to improve HDMTX clearance. Subsequent efforts will assess whether acting on such risk factors can improve MTX clearance and shorten LOS.

## Introduction

Methotrexate (MTX), a folate analog, has been used to treat malignancies since 1948 (1). The use of high dose methotrexate (HDMTX), defined as doses >500 mg/m2, is associated with excellent outcomes in childhood acute lymphoblastic leukemia (ALL) (1), and osteosarcoma (2). Despite its efficacy, HDMTX causes significant toxicity including hepatotoxicity, myelosuppression, mucositis, leukoencephalopathy, and nephrotoxicity (3).

Nephrotoxicity is common, with an incidence between 2-12% in the pediatric population (4). Acute kidney injury (5). Other factors affecting methotrexate clearance include its serum concentration, urine pH, intravascular volume, co-administration of weak acids, polymorphisms of SLCO1B1, extravascular fluid collections, and delays in recognizing toxicity and initiating treatment (3).

Conventionally, HDMTX is delivered in the inpatient setting. The development of supportive care guidelines such as vigorous intravenous (IV) hydration, alkalinization of the urine, and the use of leucovorin has considerably reduced associated morbidity (5, 6). Furthermore, the ability to monitor serum methotrexate levels during the hospitalization allows for timely interventions which may optimize clearance and reduce acute toxicity. Supportive care guidelines have been established targeting specific timeframes where optimal methotrexate exposure and clearance from the body has been achieved, which ideally can lead to predictable hospitalization durations and reductions in healthcare costs. Despite these advances, the administration of HDMTX continues to be associated with substantial toxicity, and persistent problems with delayed clearance - suggesting that additional factors may be influencing the outcome (7).

In this study, we examined pediatric oncology patients who received inpatient HDMTX. Our goal was to identify modifiable risk factors for delayed clearance (DC) which can be used to optimize methotrexate delivery and achieve timely clearance.

## Materials and methods

This study was approved by the Institutional Review Board of the Washington University School of Medicine Human Research Protection Office. We conducted a retrospective chart review of existing medical record data on pediatric oncology patients, treated at St. Louis Children’s Hospital, who received HDMTX between January 1, 2010, and December 31, 2017. The population consisted of patients with one of two diagnoses: 1) Leukemia, receiving 5 g/m2, and 2) Osteosarcoma, receiving 12 g/m2 of MTX.

The primary outcome measure was delayed clearance, defined as failure to achieve the targeted serum value of <1uM by 48 hours for patients with leukemia, and 72 hours for patients with osteosarcoma. Secondary outcome measures included 1) time to clearance (TTC) in hours, which was defined as the time from completing the infusion until achieving a serum MTX concentration <1µM, and 2) length of stay (LOS) in days, which was defined as the time interval between the documented admission time through the documented discharge time for each HDMTX encounter.

Risk factors for DC, prolonged TTC, and LOS included diagnosis, demographics, MTX doses, all MTX levels obtained, renal toxicity (defined in this study as an increase of 50% or greater in a patient’s serum Creatinine (Cr) after MTX administration), concurrent IV Supportive Care Medication (SCM), used (ondansetron, metoclopramide, lorazepam, and leucovorin), IV hydration and administration details, and urine output. Data were collected from all relevant patient encounters; those with missing key variables were omitted from the analysis.

### HDMTX administration

At our institution, HDMTX is administered by continuous IV infusion in the inpatient setting. Patients receive a 20 mL/kg Normal Saline (NS) bolus followed by standard pre-hydration at 125mL/m2/hr of ¼ NS with 30mEq NaHCO_3_ until they meet urine parameters to begin administration of HDMTX (Specific gravity </= 1.010, pH >/= 7). HDMTX for all patients is mixed in the same base fluid as pre hydration, and is then administered at the same fluid rate, 125mL/m2/hr, for 4 hours (osteosarcoma) or 24 hours (leukemia). During the time of administration and the period following, patients receive antiemetic supportive care medications (SCM) including PO or IV ondansetron, metoclopramide, diphenhydramine, and lorazepam as needed. Apart from lorazepam, all the antiemetics are incompatible with HCO_3_ and require pausing the infusion between 15-30 minutes for each administration. Additionally, patients with urine output (UO) < 2mL/kg/hr (assessed every 4 hours) may receive additional NS IVF boluses or IV furosemide at the physician’s discretion.

When the infusion is complete, post-hydration with the same base fluid is immediately started at 125mL/m2/hr. MTX levels are obtained 24 hours post infusion, and every 24 hours after until clearance (<0.1 µM). Patients also receive 15mg/m2 of IV (if > 25mg) or PO (if < 25mg) leucovorin every 6 hours, starting 24 hours (or 42 hours in some ALL COG protocols) after the HDMTX start time. Following institutional guidelines, the rate of IV fluids, as well as the dose and frequency of leucovorin, are increased if the patients experience a >25% rise in their Cr or if they exceed the expected MTX clearance threshold for time. Patients are discharged when their serum MTX concentration is <0.1 µM/L.

### Statistical analysis

SAS version 9.4 (SAS Institute, Cary, NC) was used to perform all statistical analysis. Demographic and clinical characteristics were summarized by descriptive statistics. Both univariate and multivariate generalized linear models (GLMs) with repeated measures were applied to examine the association between outcomes and risk factors. Variables examined for both univariate and multivariate analysis included: diagnosis, (leukemia vs osteosarcoma), gender, race, age at initiation of HDMTX course, weight, MTX Bag dose, MTX serum level, (24 and 48 hours), renal toxicity, IV SCM (analyzed both individually and together) day 1, 2, and 3 of the course, total IVF intake (L/m2/day and total volume) for day 1,2, 3, urine output (ml/kg/hr) on day 1, 2, 3.tp

A generalized estimating equation (GEE) approach was used to estimate the model parameters, assuming an exchangeable covariance structure, to account for within-patient dependencies. For multivariate analyses, variable selections were conducted on the fixed effects via stepwise procedures, using a significance level of 0.05 as both the entry and stay criteria.

The binary outcome, DC (delayed vs. not delayed), was modeled via logistic regression. Regression coefficients were converted to odds ratios (ORs) with 95% confidence interval (95% CI) to represent the odds that DC will occur given a particular level of risk factor, compared to the odds of DC occurring in the reference level of that risk factor.

The continuous outcomes TTC and LOS, were modeled using Poisson regression (log-linear model), given the right-skewed nature of the distribution. Regression coefficients were converted to risk ratios (also known as relative risks, RRs) with 95% CI to represent the ratio of the TTC or LOS for a given level of a risk factor with the reference level of that factor.

## Results

**Figure 1:**
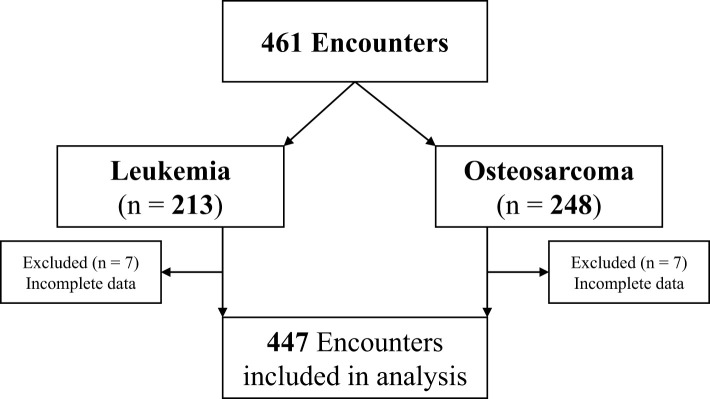
Consort diagram.

The charts of 84 patients were identified, and a total of 461 HDMTX encounters were reviewed. Of 461 unique encounters, 447 (97%) HDMTX administrations (206 leukemia, 241 osteosarcoma) were included in the analysis. Fourteen (3%) encounters (7 leukemia, 7 osteosarcoma) were excluded due to incomplete data (**Figure 1**). **Table 1** summarizes characteristics of patients included, which are representative of the general pediatric cancer population treated at this institution – predominantly white (68%), male (58.3%), and > 10 years old (66.7%).

**Table 1:** Characteristics of Patients Analyzed.

Characteristic, n (%)	Patients (Total=84)	Encounters (Total=447)
Age Group (years)		
<5	16 (19.0)	
5-10	12 (14.3)	
10-15	25 (29.8)	
≥ 15	31 (36.9)	
Diagnosis		
Osteosarcoma	27 (32.1)	241 (54.0)
Leukemia	57 (67.9)	206 (46.0)
Gender		
Male	49 (58.3)	
Female	35 (41.7)	
Race		
White	68 (81.0)	
Non-White	16 (19.0)	
Delayed Clearance		
**Yes**		357 (79.9)
Osteosarcoma		170 (47.6)
Leukemia		187 (52.4)
**No**		90 (20.1)
Osteosarcoma		71 (78.9)
Leukemia		19 (21.1)

DC of HDMTX was observed in 52% of leukemia administrations and 48% of osteosarcoma administrations. At 3 days, only 50% of all patients had achieved targeted clearance. The median TTC and LOS for patients with normal clearance were 2.5 (Q1 – Q3, 2.5 - 2.9) and 2.8 days (Q1 – Q3, 2.6 – 3.0), compared to 3.5 (Q1 – Q3, 3.0 – 4.5) and 3.7 days (Q1 – Q3, 3.1 - 4.7) for those with DC, respectively.

### Delayed clearance

Univariate analysis revealed that a diagnosis of leukemia was significantly associated with delayed clearance (OR 4.04 95% CI 1.86-8.78, p=0.0004). Gender, race, age, and weight were not associated with an increased risk of DC. Levels of MTX at 24 hours (OR 1.02 95% CI 1.01-1.03, p=0.006), and the total number of IV medications administered on day 3 (OR 1.1 95% CI 1.02-1.19, p=0.01) were associated with DC (**Table 2**). Furthermore, patients receiving a lower total dose of IV MTX (OR 0.92 95% CI 0.88-0.97, p=0.0009), and those with increased total IV intake in the first 48 hours (OR 0.91, 95% CI 0.88-0.95, p<0.0001) were less likely to develop DC.

**Table 2:** Univariate regressions with repeated measures assessing risk factors for delayed clearance, time to clearance (days), and length of stay (days).

Risk Factors	Delayed Clearance		Time to Clearance		Length of Stay	
	Odds Ratio (95% CI)	*p*-value	Risk Ratio (95% CI)	*p*-value	Risk Ratio (95% CI)	*p*-value
**Diagnosis (Leukemia vs. Osteosarcoma)**	**4.04 (1.86-8.78)**	**0.0004**	0.93 (0.81-1.07)	0.3234	1.09 (0.89-1.33)	0.3873
**Gender (Female vs. Male)**	0.89 (0.4-2)	0.7845	1.04 (0.88-1.23)	0.6191	1.24 (0.97-1.59)	0.0808
**Race (Non-White vs. White)**	0.45 (0.17-1.16)	0.0978	1 (0.78-1.28)	0.9931	1.03 (0.7-1.51)	0.8849
**Age at Methotrexate Bag 1 Start (years)**	1 (0.93-1.08)	0.9525	**1.03 (1.01-1.04)**	**<.0001**	**1.03 (1.01-1.04)**	**<.0001**
**Weight (kg)**	1 (0.99-1.02)	0.6016	**1.003 (1.0001-1.006)**	**0.0405**	1.003 (0.999-1.01)	0.1602
**MTX Bag 1 Dose (g)**	**0.92 (0.88-0.97)**	**0.0009**	1.01 (0.998-1.02)	0.1225	1 (0.98-1.01)	0.6119
**Methotrexate 24 hour**	**1.02 (1.01-1.03)**	**0.0062**	1 (1.000-1.003)	0.0559	**1.002 (1.0001-1.004)**	**0.0423**
**Methotrexate 48 hour**	NE	NE	**1.03 (1.01-1.05)**	**0.0002**	**1.03 (1.02-1.05)**	**<.0001**
**Renal Toxicity (Rise in Cr)**	1 (0.99-1.02)	0.6969	**1.005 (1.002-1.007)**	**0.0004**	**1.006 (1.002-1.01)**	**0.0012**
**IV Lorazepam Day 1**	1.24 (0.84-1.84)	0.2778	1.02 (0.94-1.1)	0.7084	0.99 (0.92-1.06)	0.7603
**IV Lorazepam Day 2**	1.02 (0.77-1.35)	0.8827	1.06 (0.97-1.17)	0.1789	1.07 (0.95-1.2)	0.2435
**IV Lorazepam Day 3**	1.17 (0.67-2.04)	0.5815	**1.13 (1.04-1.22)**	**0.0037**	**1.13 (1.03-1.23)**	**0.0079**
**IV Metoclopramide Day 1**	2.23 (0.64-7.73)	0.2061	**1.19 (1.07-1.32)**	**0.0014**	**1.2 (1.03-1.41)**	**0.0209**
**IV Metoclopramide Day 2**	1.36 (0.82-2.25)	0.2368	**1.12 (1.07-1.17)**	**<.0001**	**1.11 (1.05-1.17)**	**0.0002**
**IV Metoclopramide Day 3**	1.6 (0.81-3.16)	0.1778	**1.13 (1.08-1.18)**	**<.0001**	**1.1 (1.05-1.16)**	**0.0002**
**IV Ondansetron Day 1**	1.07 (0.72-1.59)	0.7253	0.96 (0.92-1.01)	0.0944	0.94 (0.86-1.01)	0.1007
**IV Ondansetron Day 2**	1.01 (0.82-1.25)	0.9197	0.97 (0.93-1.01)	0.1206	0.99 (0.94-1.04)	0.637
**IV Ondansetron Day 3**	1.19 (0.99-1.41)	0.0578	**1.05 (1.01-1.08)**	**0.0079**	**1.06 (1.02-1.1)**	**0.0045**
**IV Leucovorin Day 2**	0.88 (0.73-1.07)	0.2066	1.03 (0.998-1.06)	0.068	1.01 (0.97-1.05)	0.6894
**IV Leucovorin Day 3**	1.06 (0.93-1.2)	0.3735	**1.04 (1.02-1.07)**	**0.0002**	**1.04 (1.01-1.06)**	**0.0029**
**Total IV SCM Day 1**	1.26 (0.96-1.64)	0.096	1.02 (0.98-1.07)	0.3021	1.01 (0.94-1.08)	0.8004
**Total IV SCM Day 2**	0.99 (0.9-1.09)	0.8992	**1.03 (1.01-1.05)**	**0.012**	1.02 (0.998-1.05)	0.075
**Total IV SCM Day 3**	**1.1 (1.02-1.19)**	**0.0135**	**1.04 (1.03-1.06)**	**<.0001**	**1.04 (1.03-1.05)**	**<.0001**
**Total IV Intake Day 1 (L/m2/day)**	0.76 (0.51-1.13)	0.1734	1.02 (0.95-1.09)	0.5686	0.98 (0.89-1.08)	0.7189
**Total IV Intake Day 2 (L/m2/day)**	**0.91 (0.88-0.95)**	**<.0001**	1 (0.99-1.01)	0.7265	1 (0.99-1.01)	0.8091
**Total IV Intake Day 3 (L/m2/day)**	1.44 (0.97-2.12)	0.0699	**1.19 (1.14-1.24)**	**<.0001**	**1.16 (1.09-1.22)**	**<.0001**
**Total IV Intake Day 1 (L)**	0.89 (0.71-1.13)	0.3472	**1.05 (1.02-1.08)**	**0.0027**	1.03 (0.99-1.07)	0.1133
**Total IV Intake Day 2 (L)**	**0.96 (0.95-0.98)**	**<.0001**	1.01 (0.995-1.02)	0.2934	1 (0.99-1.02)	0.5138
**Total IV Intake Day 3 (L)**	1.18 (0.98-1.41)	0.0748	**1.07 (1.05-1.1)**	**<.0001**	**1.07 (1.03-1.11)**	**0.0016**
**UOP Day 1 (mL/kg/hr)**	**0.77 (0.64-0.91)**	**0.0031**	**0.94 (0.9-0.98)**	**0.0044**	**0.92 (0.86-0.98)**	**0.0086**
**UOP Day 2 (mL/kg/hr)**	0.93 (0.79-1.1)	0.4106	0.98 (0.95-1.01)	0.16	0.97 (0.93-1.01)	0.0986
**UOP Day 3 (mL/kg/hr)**	NE	NE	0.9997 (0.995- 1.004)	0.8767	1 (0.99-1.004)	0.3391

Bold = statistically significant; NE, Not evaluable.

Multivariate analysis of demographic risk factors for DC showed that patients with a diagnosis of leukemia were seven times more likely to experience DC compared to those with a diagnosis of osteosarcoma (**Table 3**) (OR 7.64 95% CI 3.18-18.39, p<0.0001). Gender, race, age, and weight did not have a significant impact on DC. Decreased UOP on day 1 (OR 0.76, 95% CI 0.62-0.92, p=0.005), and increased IVF intake on day 3 (OR 1.74, 95% CI 1.18-2.58, p=0.006) were associated with DC.

**Table 3:** Multivariate regressions with repeated measures assessing risk factors for delayed clearance, time to clearance (days), and length of stay (days)*.

Risk Factors	Delayed Clearance		Time to Clearance		Length of Stay	
	Odds Ratio (95% CI)	*p*-value	Risk Ratio (95% CI)	*p*-value	Risk Ratio (95% CI)	*p*-value
**Diagnosis (Leukemia vs. Osteosarcoma)**	7.64 (3.18-18.39)	<.0001				
**Age at Methotrexate Bag 1 Start (years)**			1.022 (1.01-1.032)	<.0001	1.07 (1.03-1.108)	0.0001
**Weight (kg)**					0.992 (0.985-0.999)	0.0226
**Methotrexate 24 hour**			1.001 (1.0003-1.002)	0.0123	1.001 (1.0002-1.003)	0.0256
**Methotrexate 48 hour**			1.024 (1.01-1.037)	<.0001	1.029 (1.02-1.041)	<.0001
**Renal Toxicity (Rise in Cr)**			1.004 (1.003-1.006)	<.0001	1.006 (1.003-1.008)	<.0001
**IV Lorazepam Day 3**			1.08 (1.03-1.132)	0.0011		
**IV Metoclopramide Day 3**			1.086 (1.05-1.127)	<.0001		
**IV Leucovorin Day 2**					0.938 (0.901-0.976)	0.0017
**Total IV SCM Day 3**					1.042 (1.03-1.058)	<.0001
**Total IV Intake Day 3 (L/m2/day)**	1.74 (1.18-2.58)	0.0057	1.094 (1.05-1.137)	<.0001		
**UOP Day 1 (mL/kg/hr)**	0.76 (0.62-0.92)	0.0046				

^*^The multivariate models were fitted on risk factors selected via stepwise process using a significant level of 0.05 as both the entry and stay criteria. The table exclusively presents statistically significant findings.

### Time to clearance

Univariate analysis demonstrated that increased weight (RR 1.003, 95% CI 1.0001-1.006, p=0.040) and age (RR 1.03, 95% CI 1.01-1.04, p<0.0001) were associated with prolonged TTC, while other demographic factors such as diagnosis, gender, and race were not. The use of IV metoclopramide at any time during hospitalization (d1 RR 1.19, p=0.0014**;** d2 RR 1.12, p<0.0001**;** d3 RR 1.13, p<0.001) were both associated with increased TTC. All antiemetics were found to be associated with increased TTC on day 3; however, almost all patients present on day 3 and beyond by definition had DC (>48 hours for leukemia, >72 hours for osteosarcoma), thus patients in the hospital beyond day three already fulfill criteria of delayed clearance. The total number of IV medications on day 2 (RR 1.03, 95% CI 1.01-1.05, p=0.01), day 3 (RR 1.04 95% CI 1.03-1.06, p<0.0001), and the use of IV leucovorin on day 3 (RR=1.04, 95% CI 1.02-1.07, p=0.0002) were also associated with increased TTC. Increased UO on day 1 was protective against increased TTC on univariate analysis (RR 0.94, 95% CI 0.9-0.98, p=0.004).

On multivariate analysis, the only demographic factor associated with increased TTC was age (RR 1.02, 95% CI 1.01-1.03, p<0.0001), while diagnosis, gender, race, and weight were not. Other factors associated with increased TTC include serum levels of MTX at 24 (RR 1.001, 95% CI 1.0003-1.002, p=0.012) and 48 hours (RR 1.024, 95% CI 1.01-1.04, p<0.0001); RT (RR 1.004, 95% CI 1.003-1.006, p<0.0001); the increased use of IV lorazepam (RR 1.08, 95% CI 1.03-1.13, p=0.001), IV metoclopramide (RR 1.09, 95% CI 1.05-1.13, p<0.0001), and IV fluids(L/m^2^/day) (RR 1.09, 95% CI 1.05-1.14, p<0.0001) on day 3.

### Length of stay

Similar to TTC, increasing age was significantly associated with prolonged LOS on univariate analysis (RR 1.03, 95% CI 1.01-1.04, p<0.0001). Other demographic factors did not significantly impact LOS. The use of IV metoclopramide on any day (d1 RR 1.2, p=0.02**;** d2 RR 1.1, p=0.0002**;** d3 RR 1.1, p=0.0002), use of IV lorazepam on day 3 (RR 1.13 95% CI 1.03-1.23, p=0.008), total IV medications on day 3 (RR 1.04, 95% CI 1.03-1.05, p<0.0001), and total IV fluid intake (L/m^2^/day) on day 3 (RR 1.16, 95% CI 1.09-1.22, p<0.0001) were all associated with increased LOS. Like TTC, increased UO on Day 1 was protective against increased LOS (RR 0.92, 95% CI 0.86-0.98, p=0.009).

Multivariate analysis of showed that age (RR 1.07, 95% CI 1.03-1.108, p=0.0001), and weight (RR 0.99, 95% CI 0.985-0.999, p=0.02) were the only two demographic factors associated with prolonged LOS. MTX levels at 24 (RR 1.001, 95% CI 1.0002-1.003, p=0.0256) and 48 hours (RR 1.03, 95% CI 1.02-1.04, p<0.0001); RT (RR 1.006, 95% CI 1.003-1.008, p<0.0001), and total IV medications on day 3 (RR 1.04, 95% CI 1.03-1.06, p<0.0001) were all associated with increased LOS. The use of leucovorin on day 2 (RR 0.94, 95% CI 0.90-0.98, p=0.002) was correlated with a shorter LOS.

## Discussion

This retrospective analysis demonstrates that, despite following standard MTX administration protocols, most of our patients receiving HDMTX experienced DC, a prolonged TTC, and increased LOS. The following modifiable risk factors were identified: 1) UO in the first 24 hours had a significant impact on DC, TTC and LOS, 2) renal toxicity was associated with a prolonged TTC and LOS, and 3) The use of anti-emetic medications in the first two days, particularly metoclopramide, was correlated with increased TTC and LOS.

Many of the variables that were significant in one outcome but not in another were still associated with delayed clearance of HDMTX but failed to achieve statistical significance. Varying hydration rates (based on age, body surface area (BSA), clearance history), and SCM administration likely contributed to this discrepancy. DC is also different for leukemia versus osteosarcoma since their targeted time to clearance is different. TTC may be better indicator for difficulties with MTX clearance, as TTC is independent from the targeted time to achieve a MTX level sufficient for discharge from the hospital. Furthermore, factors influenced on day 3 and beyond may reflect challenges experienced with MTX administration in the previous two days and may select for patients destined to have DC, increased TTC and prolonged LOS. Consequently, many of these variables failed to maintain their significance in multivariate analysis.

In trying to define a unifying theme, we propose that there are two clinical activities that may represent the overriding influence on the outcome measures: 1) High concentrations of MTX in the urine leading to crystallization and prerenal azotemia (reflected by high 24 and 48 hour levels of MTX, and RT) and 2) Suboptimal IVF hydration leading to alterations in UO, influencing clearance of MTX (reflected by UO, IVF, and the necessary interruptions in IVF hydration to administer scheduled SCM such as metoclopramide).

Early studies have demonstrated that the severity of toxicities associated with HDMTX is exacerbated by higher serum concentrations of the drug, and prolonged exposure (8). This observation highlights the importance of optimizing HDMTX administration in ways that maintain desired serum concentrations and promote timely clearance. Thus, continuing efforts to identify modifiable risk factors is valuable as it could lead to further alterations in practice - potentially resulting in improved, predictable, and consistent clearance of HDMTX.

Decreased urine output on day 1 and decreased IV fluid intake on day 2 were associated with delayed clearance. This observation suggests that hydration strategies could be altered to optimize IV intake and UO within the first 48 hours. Administration of higher rates of IVF prior to initiation of HDMTX and continuing throughout the hospitalization could demonstrate the protective effects of increased urine output in the first day and total fluids on the second day to provide timely clearance of HDMTX. Nakano et al. reported similar findings within their institutional cohort where they found that a lower urine volume per body surface area on day 1 and 2, as well as a lower GFR were significant risk factors for DC (9). Further investigation is needed to identify the ideal hydration strategy to achieve optimal clearance.

MTX is primarily renally excreted. The concurrent use of some medications has been shown to significantly alter serum and urine pH, causing impaired MTX clearance due to crystal formation, or acute kidney injury (3, 10). Our findings suggest a more subtle relationship between IV medication administration and DC. Our institutional practice for access in patients with acute lymphoblastic leukemia and osteosarcoma is placement of single lumen port-a-catheters. Hence, IV SCM necessitate the interruption of IVF due to the incompatibility of those medications with sodium bicarbonate contained within our methotrexate infusion and post infusion IVF. We hypothesize that DC may be linked to the frequent and necessary interruption in IV hydration to administer SCM. In our retrospective analysis we have found that patients with a diagnosis of leukemia were 7 times more likely to experience DC, we believe this could potentially be the result of a longer MTX infusion time (24 hours, compared to 4 hours for osteosarcoma), leading to more frequent, prolonged, interruptions in hydration during the infusion to administer SCMs. Given that MTX infusions must be completed within 24 hours, increasing the MTX infusion rate to account for lost time is also likely contributing to delayed clearance by altering the desired AUC. Careful consideration of IVF components is necessary given that MTX stability, metabolization, and clearance are affected by changes in pH (5). Previous investigators found that higher sodium content in hydration fluids was significantly associated with a lower serum MTX at 24 hours; supporting the hypothesis that IV fluid composition influences MTX levels and consequently clearance (11).

Because SCM interrupt our standard hydration, a potential intervention would be altering IV fluid composition to optimize medication compatibility and reduce interruptions. Patients receiving HDMTX in sodium acetate, as opposed to sodium bicarbonate, have had comparable clearance patterns without increased toxicity, as reported previously in an institutional trial (12). Sodium acetate is compatible with most IV anti-emetics and leucovorin, eliminating the need for frequent pauses in hydration. Alternative strategies could include the utilization of oral and long-acting anti-emetics, such as aprepitant.

Given the role renal function plays in MTX clearance, ensuring adequate renal function prior to MTX administration is necessary to reduce iatrogenic toxicity (5). Serum Cr and Cr clearance (CrCl) influence serum concentrations of MTX and its subsequent clearance (13). A rise in serum Cr above baseline indicates renal dysfunction, decreased GFR, and results in delayed MTX clearance (14). In our patient cohort, we found that a rise in Cr (>50%) was significantly associated with prolonged TTC and LOS. The strategies described above to maintain high rates of IVF hydration may result in reduced incidence of renal impairment. A retrospective analysis showed that increasing hydration rates in patients with a history of DC was associated with a statistically significant decrease in serum Cr levels at 24, 42, and 48 hours (15). Furthermore, when patients received increased hydration, a history of DC was no longer a significant risk factor for subsequent delays.

Refinements in monitoring serum creatinine and providing prompt interventions to address acute changes in serum creatinine could further ensure optimal HDMTX clearance. Prior studies examining variables that influence HDMTX clearance have provided alternative results. Some studies demonstrated a statistically significant effect of GFR on clearance (16), and even suggested that renal function is a better predictor of toxicity compared to serum levels of MTX (13). Other studies did not support these findings (17). The validity of our results will be dependent upon the success of altering the modifiable risk factors described in this report to improve HDMTX clearance and provide a predictable LOS in the face of minimizing renal dysfunction.

An unexpected result found in our analysis was the association between IV leucovorin use on day 2 and a shorter LOS of multivariate analysis. Leucovorin rescue, standardly 15mg/m^2^ of a 1mg/mL IV product is given over 15 minutes. The IV fluids used for hydration during HDMTX are typically Dextrose 5% and 0.25 normal saline with 30 mEq of sodium bicarbonate given at a standard rate of 125 ml/m^2^/hr. Leucovorin, standardly reconstituted in saline, is given every 6 hours. However, it is given more frequently and at higher doses in patients experiencing delayed clearance. This results in a total infusion time of 120 minutes/day- where hydration must be paused due to incompatibility of leucovorin with the sodium bicarbonate containing fluid. During that time, normal saline fluids are utilized for hydration. We propose that the theoretical benefit of increasing the frequency of IV leucovorin administration may be due to an increase in fluid hydration with saline, with its higher sodium concentration and isotonic composition, effectively increasing intravascular volume and improving renal perfusion resulting in enhanced MTX clearance (11).

This report is one of the most comprehensive analyses of HDMTX administration to date, with an attempt to include all variables which may have impacted the clearance of MTX and subsequently the TTC and LOS. Our discussion of how the significant variables impacted the outcome, was validated by interviewing staff and thus we eliminated speculation on how some variables influenced the outcome and allowed us to consolidate the results to generate proposed interventions/enhancements which we will test to demonstrate that they can lead to improvements in the administration of this important agent.

MTX is mainly cleared through renal excretion, making kidney function and hydration the most critical factors for its clearance. Although genetic polymorphisms, such as those in the genes SLC19A1, which encodes for the reduced folate carrier, and the MTHFR gene, and SCLO1B1, can contribute to variations in MTX metabolism (18), their effects are seemingly overshadowed by kidney function. For example, the GFR is a primary determinant for MTX pharmacokinetics and can significantly alter the drug’s clearance rate (13). Similarly, intravenous fluid intake can markedly enhance MTX renal clearance by dilution and facilitation of excretion. Hence, while specific genetic polymorphisms can modulate MTX metabolism, their clinical impact appears to be less than that of renal function and hydration status. Multiple Machine Learning (ML) algorithms designed to predict MTX clearance have incorporated specific genetic polymorphisms into their risk prediction models, with only modest improvement in the accuracy of these models (10). The better performing and freely available model aimed at predicting serum MTX levels and the need for glucarpidase used a large patient cohort and achieved high accuracy by incorporating variables such as MTX dose, serum levels, BSA, weight, age, and creatinine, without genetic polymorphisms (19); suggesting that even though genetic polymorphisms play a role in HDMTX clearance, for most patients, other variables such as dose, weight, creatinine clearance and subsequently GFR play a greater role in MTX pharmacokinetics and clearance; thus, these factors would benefit from further optimization.

MTX also has the potential for pharmacokinetic interactions with other medications that can influence its clearance. Antiemetics like ondansetron and metoclopramide are frequently administered alongside MTX to manage chemotherapy-induced nausea and vomiting. While these antiemetics do not significantly interfere with MTX clearance, it is essential to monitor for potential additive renal toxicity. Lorazepam, often used for anxiety and nausea in chemotherapy settings, also shows no substantial interaction with MTX clearance (20). However, any drug affecting renal function or competing for renal tubular secretion could theoretically alter MTX pharmacokinetics. We found that many of these anti-emetics were individually and collectively associated with increased TTC and LOS; however, as previously stated, we believe this effect is due to persistent interruptions in IV hydration rather than the individual medications.

Our study is limited by its retrospective nature, data from a single center, and potentially non-universal institutional administration practices. Variations in the preparation and administration of IV chemotherapy, fluids, and medications by individuals could not be ruled out. Additionally, institutional differences in HDMTX protocols, including variations in IV fluid composition, rate, and concomitant medication use, may restrict the generalizability of our findings.

Despite the limitations, we identified testable modifiable risk factors which could influence MTX clearance. Further studies optimizing IVF hydration, supportive care medication administration, and prompt interventions to address RT could significantly improve the administration of HD-MTX, reducing toxicity, delayed clearance, and optimizing hospital LOS.

## Data availability statement

The original contributions presented in the study are included in the article/supplementary material. Further inquiries can be directed to the corresponding author.

## Ethics statement

The studies involving humans were approved by The Institutional Review Board (IRB) at Washington University in St Louis. The studies were conducted in accordance with the local legislation and institutional requirements. Written informed consent for participation in this study was provided by the participants’ legal guardians/next of kin.

## Author contributions

EM: Data curation, Formal analysis, Investigation, Supervision, Validation, Writing – original draft, Writing - review & editing. SS: Conceptualization, Data curation, Investigation, Methodology, Writing – review & editing. DW: Data curation, Investigation, Validation, Writing – review & editing. NW: Data curation, Formal Analysis, Investigation, Software, Validation, Writing – review & editing. RM: Writing – review & editing. RH: Conceptualization, Formal Analysis, Investigation, Project administration, Supervision, Writing – review & editing, Methodology, Validation.
