# Corrigendum: Factors influencing delayed clearance of high dose methotrexate (HDMTX) in pediatric, adolescent, and young adult oncology patients

**DOI:** 10.3389/fonc.2023.1333273

**Published:** 2023-11-27

**Authors:** Ema Mosleh, Stacy Snyder, Ningying Wu, Daniel N. Willis, Rema Malone, Robert J. Hayashi

**Affiliations:** ^1^ Department of Pediatrics, Washington University School of Medicine, St. Louis, MO, United States; ^2^ Biostatistics Shared Resource, Division of Public Health Sciences and Siteman Cancer Center, Department of Surgery, Washington University School of Medicine, St. Louis, MO, United States; ^3^ Division of Pediatric Hematology/Oncology, St. Louis Children’s Hospital, St. Louis, MO, United States

**Keywords:** methotrexate, HDMTX, supportive care, delayed clearance, nephrotoxicity, adverse effect, pediatric cancer, AYA

In the original article, there was an error in the **author list order**, as published. Instead of “Ema Mosleh^1*^, Stacy Snyder^1†^, Ningying Wu^2^, Daniel N. Willis^1^, Robert J. Hayashi^1^, Rema Malone^3^”, It should be “Ema Mosleh^1*^, Stacy Snyder^1†^, Ningying Wu^2^, Daniel N. Willis^1^, Rema Malone^3^, Robert J. Hayashi^1^”.

The authors apologize for these errors and state that they do not change the scientific conclusions of the article in any way. The original article has been updated.

